# Exploring the protease inhibitory potential of 2-bromo-2-nitro-1,3- propanediol (Bronopol): Initial findings

**DOI:** 10.1016/j.bbrep.2026.102791

**Published:** 2026-09-17

**Authors:** Chao Jiang, Gary Krzyzanowski, M. Rohan Fernando

**Affiliations:** Molecular Diagnostic Research Laboratory, Center for Sensory Neuroscience, Boys Town National Research Hospital, Omaha, NE, 68131, USA

**Keywords:** Protease inhibitors, Proteases, Bronopol, 2-Bromo-2-nitro-1,3-propanediol, Proteinase K, Trypsin, Papain, Pepsin, BSA, Ovalbumin

## Abstract

The compound 2-bromo-2-nitropropane-1,3-diol also known as Bronopol (BP) is a widely used antimicrobial preservative which highly soluble in water. Although its antimicrobial mechanism has been well described, its potential to modulate protease activity has not been previously examined. Proteases represent one of the largest and most industrially important classes of enzymes, and protease inhibitors are essential tools in research, biotechnology, and therapeutic development. In this study, we investigated the ability of BP to inhibit serine, cysteine, and aspartic proteases using both synthetic and natural proteins.

Protease inhibition was quantified using trypsin, proteinase K, papain, and pepsin. BP produced a clear dose-dependent inhibition of all proteases tested, with distinct sensitivity profiles. Proteinase K and trypsin activities declined progressively across 50–1000 μM BP, reaching approximately 20% residual activity at the highest concentration. Papain showed the greatest susceptibility, exhibiting near-complete inhibition (>95%) at 500–1000 μM. Pepsin displayed modest inhibition at low BP concentrations but substantial loss of activity above 250 μM.

Proteolytic cleavage of bovine serum albumin (BSA) and ovalbumin were assessed by SDS-PAGE. Proteinase K alone caused extensive degradation of both substrates; however, BP at 2500–10 000 μM markedly reduced proteolysis and preserved full-length proteins, confirming protective effects observed in enzymatic assays.

Together, these findings identify BP as an inhibitor of serine, cysteine, and aspartic proteases, with potent effects at moderate to high concentrations. This previously unreported activity suggests additional functional roles for BP in biochemical systems and highlights its potential relevance in research and industry.

## Highlights

• Investigated inhibition of serine, cysteine, and aspartic proteases by Bronopol.
• BP showed dose-dependent inhibition of trypsin, proteinase K, papain, and pepsin.
• BP markedly reduced proteinase K–mediated degradation of BSA and ovalbumin.
• These findings identify BP as a broad-spectrum protease inhibitor.

## 1 Introduction

Bronopol (2-bromo-2-nitropropane-1,3-diol) is a broad-spectrum antimicrobial agent widely used across industrial and scientific sectors. Developed in the 1960s, it is valued for its strong activity against bacteria, particularly Gram-negative organisms and for its stability in aqueous formulations [1,2]. BP acts by generating reactive species, including formaldehyde, which disrupt microbial enzymes and essential cellular functions [3,4]. These properties make it an effective preservative in cosmetics, personal-care products, household cleaners, adhesives, and paints. In industrial environments such as paper mills, cooling-tower systems, and oilfield operations, BP is used to control microbial fouling and biofilm formation [5]. In scientific research, BP serves as a reliable reference antimicrobial and preservative in formulation stability testing and microbial susceptibility studies [6]. Its versatility continues to support product quality and research advancement.

Proteases are a broad class of enzymes that catalyze the hydrolysis of peptide bonds, enabling the breakdown, processing, and activation of proteins. Proteases are classified by their catalytic Mechanisms. According to this classification there are seven groups of proteases namely serine proteases, cysteine proteases, aspartic proteases, metalloproteases, threonine proteases, glutamic proteases and asparagine peptide lyases. Proteases also can be classified by site of action on the Protein. This classification divides proteases into two groups, endopeptidases where protein is cleaved inside the polypeptide chain and exopeptidases where protein is cleaved from the ends of the protein. Proteases are important for protein turnover, signaling and digestion, and they now represent the largest class of industrial biocatalysts, widely used in food processing, detergents, leather treatment and bioremediation [7]. Microbial and plant proteases are especially attractive because they are efficient, biodegradable and compatible with green, low-energy processes [8]. Protease inhibitors are small molecules or peptides that inhibit the activity of proteases. These inhibitors have become indispensable in both research and industry. In basic and translational science, selective inhibitors stabilize proteins during extraction, exploring signaling pathways and serve as chemical probes for assigning protease function. Clinically, protease inhibitors strengthen many antiviral and anticancer therapies and are being intensively developed against targets such as HIV, HCV and SARS-CoV-2 proteases, as well as dysregulated human serine and cysteine proteases [9]. Their dual role as tools and drugs makes protease inhibitors central to modern biotechnology, pharmaceutical innovation and enzyme-based manufacturing across discovery, scale-up, quality control and modern product stabilization worldwide [10,11].

In this present study we explore the protease inhibitory potential of widely used antimicrobial agent BP using in vitro assay systems.

## 2 Methods and materials

### 2.1 Reagents

The Pierce Protease Assay Kit was obtained from Thermo Scientific Pierce Biotechnology (Rockford, IL, USA). Proteinase K was purchased from Gold Biotechnology (St. Louis, MO, USA), papain from Sigma-Aldrich (St. Louis, MO, USA), and pepsin from Roche (San Francisco, CA, USA). Mini-PROTEAN TGX gels (4–20%) were acquired from Bio-Rad (Hercules, CA, USA). GelCode Blue Stain Reagent, bovine serum albumin (BSA), and ovalbumin were obtained from Thermo Scientific (Waltham, MA, USA).

### 2.2 Protease activity assays

Protease activity was quantified in the presence of BP using a succinylated casein as substrate following the manufacturer's instructions for the Pierce Protease Assay Kit. Trypsin, Proteinase K, and papain activities were assessed in assay buffer composed of 50 mM borate, pH 8.5, whereas pepsin activity was measured at pH 2.0 in 10 mM HCl.

For each reaction, 50 μL of enzyme solution (25 μg/mL trypsin, 5 μg/mL Proteinase K, or 100 μg/mL papain and 30 μg/mL pepsin) containing varying concentrations of BP was added to individual wells of a 96-well microplate. Subsequently, 100 μL of assay buffer or 100 μL of succinylated casein substrate were added. Plates were incubated for 20 min at room temperature, followed by addition of 50 μL of TNBSA working solution. After an additional 20 min incubation, absorbance was measured at 450 nm using a SpectraMax iD5 plate reader (Molecular Devices). A control experiment was conducted with succinylated casein substrate without protease in the presence or absence of 1000 μM BP to study whether BP could modify the substrate. Another experiment was conducted to investigate the effect of 1000 μM BP on the reaction between primary amine and TNBSA. Glycine in assay buffer (200 μg/100 μL) was used as the source of primary amine in the presence or absence of 1000 μM BP. After incubating with TNBSA for 20 min OD 450 nm was measured.

### 2.3 Proteolytic inhibition assays

Inhibition of proteolytic activity by BP was assessed using BSA and ovalbumin as natural protein substrates at final concentrations of 0.5 mg/mL and 1 mg/mL, respectively. Substrates were incubated with 5 μg/mL Proteinase K and varying concentrations of BP for 2 days at room temperature. Reactions were terminated by addition of 5× SDS loading buffer followed by heating at 80 °C for 10 min.

Protein cleavage products were analyzed by 4–20% Tricine SDS-PAGE, and gels were stained using GelCode Blue Stain Reagent.

### 2.4 Molecular docking analysis of Bronopol against proteases

Molecular docking was performed to predict potential interaction sites between Bronopol and the proteases evaluated in vitro. The tested enzymes included trypsin, proteinase K, papain, and pepsin. Representative protein structures were obtained from the Protein Data Bank and prepared prior to docking. The structures used were trypsin, proteinase K, papain, and pepsin corresponding to PDB entries 1TRN, 1IC6, 9PAP, and 1PSN, respectively. Bronopol, also known as 2-bromo-2-nitro-1,3-propanediol, was generated from its canonical SMILES representation and converted into a three-dimensional ligand structure using RDKit. Hydrogen atoms were added and the ligand geometry was energy-minimized before conversion to PDBQT format using Meeko. Protein structures were cleaned by removing crystallographic waters and non-protein heteroatoms. Missing atoms were repaired and hydrogens were added using PDBFixer/OpenMM. Receptors were protonated at pH 7.4 for trypsin, proteinase K, and papain, while pepsin was protonated at pH 2.0 to reflect its acidic catalytic environment. Receptor PDBQT files were generated using Open Babel.

Docking was performed using AutoDock Vina. For each enzyme, two docking strategies were used: active-site-focused docking and blind docking. Active-site-focused docking boxes were centered on known catalytic residues. Blind docking boxes were generated to encompass the full protein structure, allowing assessment of whether Bronopol preferentially localized to catalytic or distal regions. The catalytic residues used for active-site-focused docking were Asp39/His69/Ser224 for proteinase K, Cys25/His159/Asn175 for papain, and Asp32/Asp215 for pepsin. For trypsin, residue numbering in the selected PDB structure did not match the canonical trypsin catalytic numbering; therefore, the PDB-local catalytic triad His40/Asp84/Ser191 was identified and used for corrected active-site docking.

Docking was performed with an exhaustiveness of 64 for active-site-focused docking and 128 for blind docking. The top-ranked docking pose for each run was selected based on the AutoDock Vina score. Residue proximity analysis was performed by identifying protein residues within 4 Å of Bronopol in the top-ranked pose. Poses were classified as catalytic-site proximal if Bronopol was located within 5 Å of one or more catalytic residues. Docking poses were visualized using PyMOL, with Bronopol shown as cyan sticks, catalytic residues as orange sticks, and nearby residues as pale green sticks. AutoDock Vina scores are reported in kcal/mol as docking scores. These values were interpreted as relative computational estimates of binding favorability and not as experimentally measured binding free energies.

### 2.5 Statistical analysis

GraphPad online Quick Calcs *t*-test calculator software was used for statistical analysis. (http://www.graphpad.com/quickcalcs/ttest1.cfm). Paired, two-tailed Student's t-test analysis was performed and p < 0.05 was considered statistically significant.

## 3 Results

**Fig. 1:**
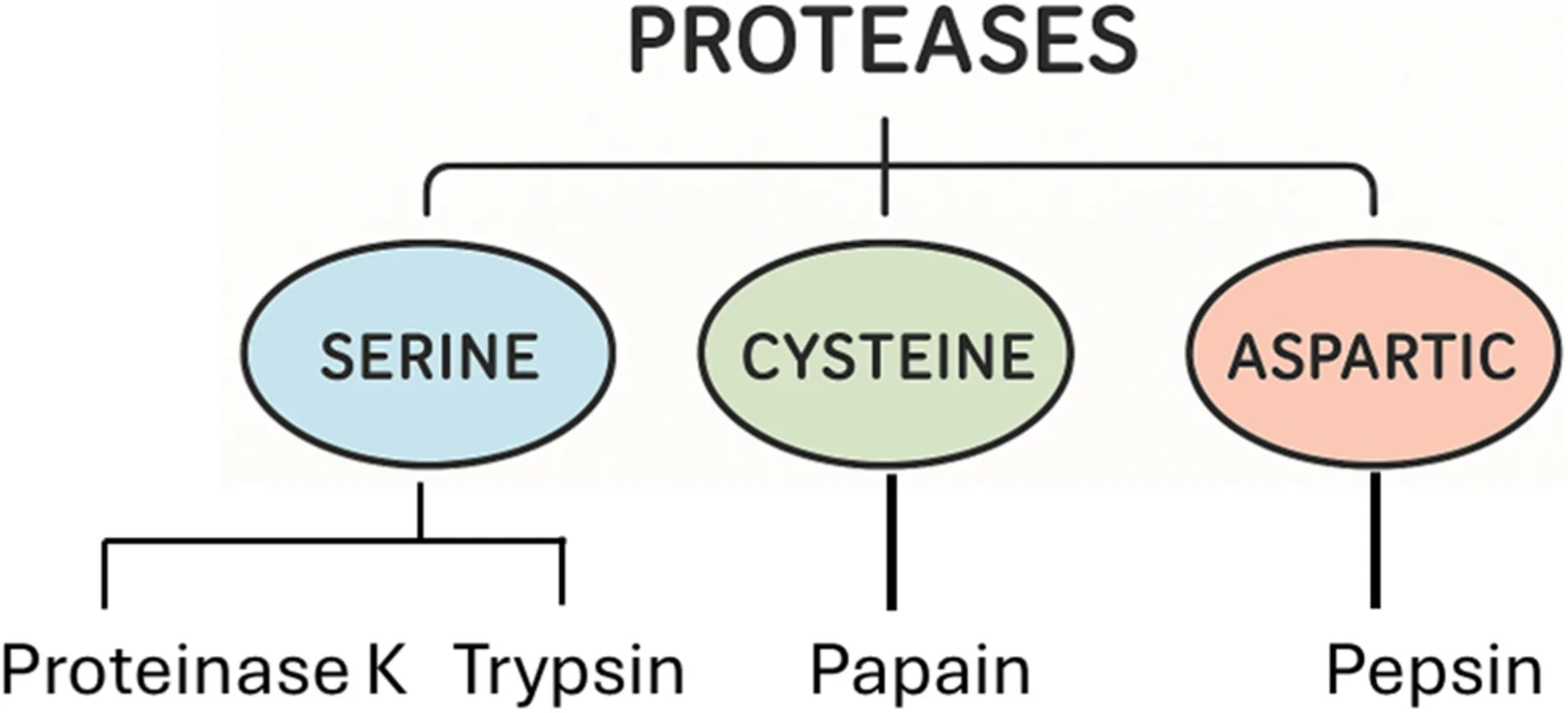
**Schematic representation of three major classes of proteases and representative examples.** Serine proteases are exemplified by proteinase K and trypsin, cysteine proteases by papain, and aspartic proteases by pepsin.

In this study we investigated the BP mediated inhibition of three groups of proteases, serine proteases, cysteine proteases and aspartic proteases (Fig. 1).

### 3.1 Assay controls

To investigate whether BP could modify the substrate succinylated casein, a control experiment was performed in the absence of protease. The substrate was incubated with 1000 μM BP for 20 min, followed by the addition of TNBSA and a further 20-min incubation before measuring absorbance at 450 nm. No absorbance was detected at 450 nm, indicating that BP did not modify the substrate (data not shown).

An additional control experiment was conducted to determine whether BP affected the reaction between primary amines and TNBSA. Glycine was used as the primary amine and incubated with or without 1000 μM BP for 20 min, followed by the addition of TNBSA and a further 20-min incubation before measuring absorbance at 450 nm. No difference in absorbance was observed between samples treated with or without BP, indicating that BP does not interfere with the reaction between primary amines and TNBSA (Supplementary Material Fig. 1).

### 3.2 Inhibition of serine proteases by BP

**Fig. 2:**
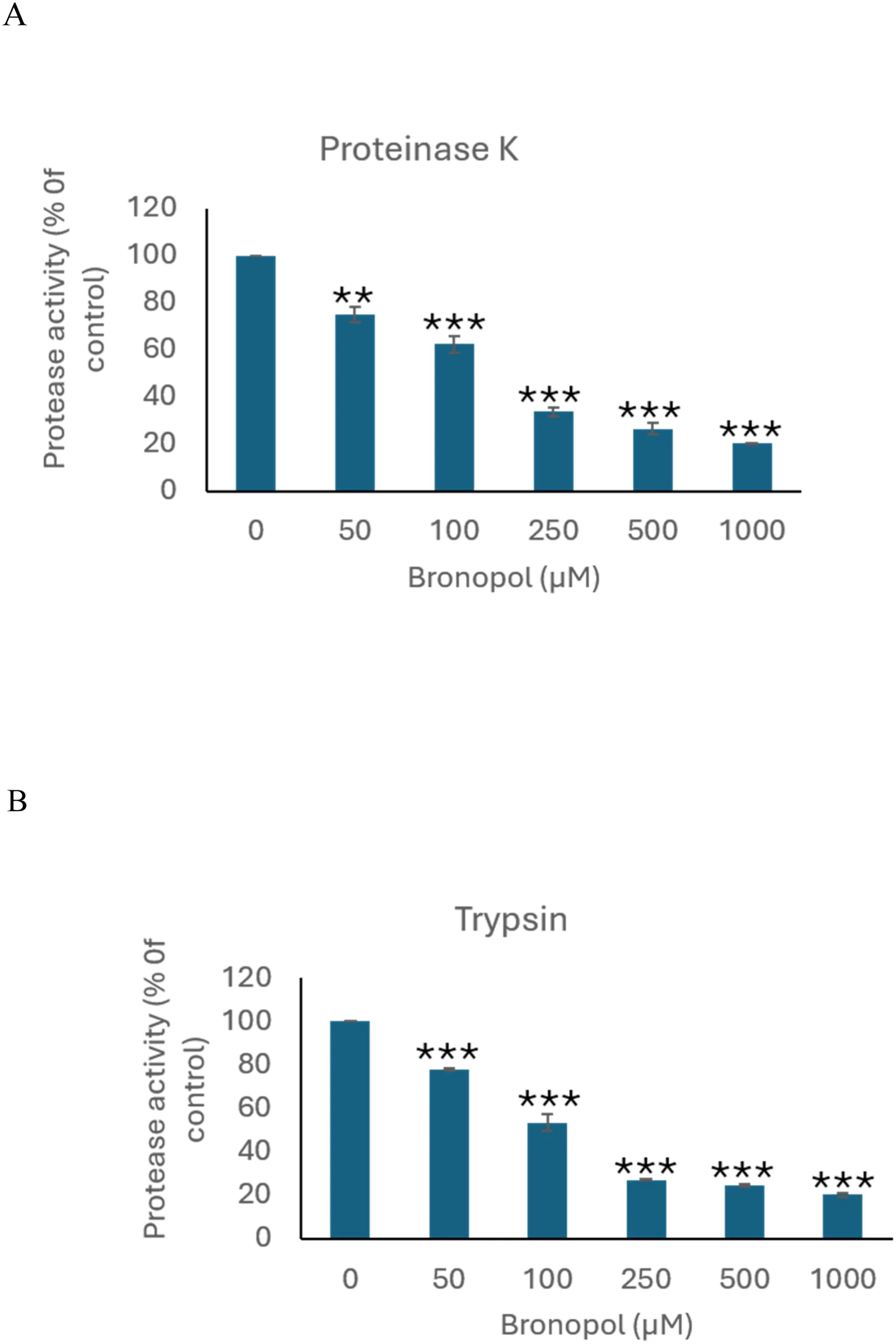
**Dose dependent inhibition of proteinase K and trypsin by BP. A**, Effect of different concentrations of BP on proteinase K activity. **B**, Effect of different concentrations of BP on trypsin activity. Protease activity was measured using the Pierce Protease Assay Kit as described in methods section. Proteinase K and trypsin concentrations in the reaction mixture were 5 μg/mL and 25 μg/mL, respectively. N = 6. *p < 0.05, **p < 0.01, ***<0.001.

Two serine proteases, trypsin and proteinase K enzymes were used to study the inhibitory effects of BP on serine proteases. The inhibitory effect of BP on Proteinase K activity was evaluated across concentrations ranging from 0 to 1000 μM. As shown in Fig. 2A, Proteinase K activity decreased progressively with increasing BP concentration. In the absence of BP, the enzyme retained full activity (100%). At 50 μM, activity declined to approximately 75%, followed by a further reduction to about 62.4% at 100 μM. More pronounced inhibition was observed at higher concentrations: activity dropped to roughly 33.6% at 250 μM and to 26.4% at 500 μM. At the highest concentration tested, 1000 μM, Proteinase K activity was reduced to approximately 20% of the control. Overall, the results demonstrate a clear dose-dependent inhibitory effect of BP on Proteinase K activity. The IC50 value for BP in this assay was calculated to be 126.6 μM (Supplementary material Fig. 2 and Table 1).

The effect of BP on trypsin activity was also assessed across concentrations ranging from 0 to 1000 μM. As shown in Fig. 2B, BP caused a progressive, dose-dependent reduction in trypsin activity. In the absence of BP, trypsin retained full activity (100%). At 50 μM, activity decreased to approximately 76.4%, followed by a further decline to around 48.9% at 100 μM. More substantial inhibition was observed at higher concentrations: trypsin activity was reduced to roughly 27% at 250 μM and to 24.8% at 500 μM. At the highest concentration tested, 1000 μM, trypsin activity declined to about 20% of the control. These results indicate that BP inhibits trypsin in a clear concentration-dependent manner, with higher BP levels leading to markedly reduced enzymatic activity. The IC50 value for BP in this assay was calculated to be 90.8 μM (Supplementary material Fig. 2 and Table 1).

### 3.3 Inhibition of cysteine proteases by BP

**Fig. 3:**
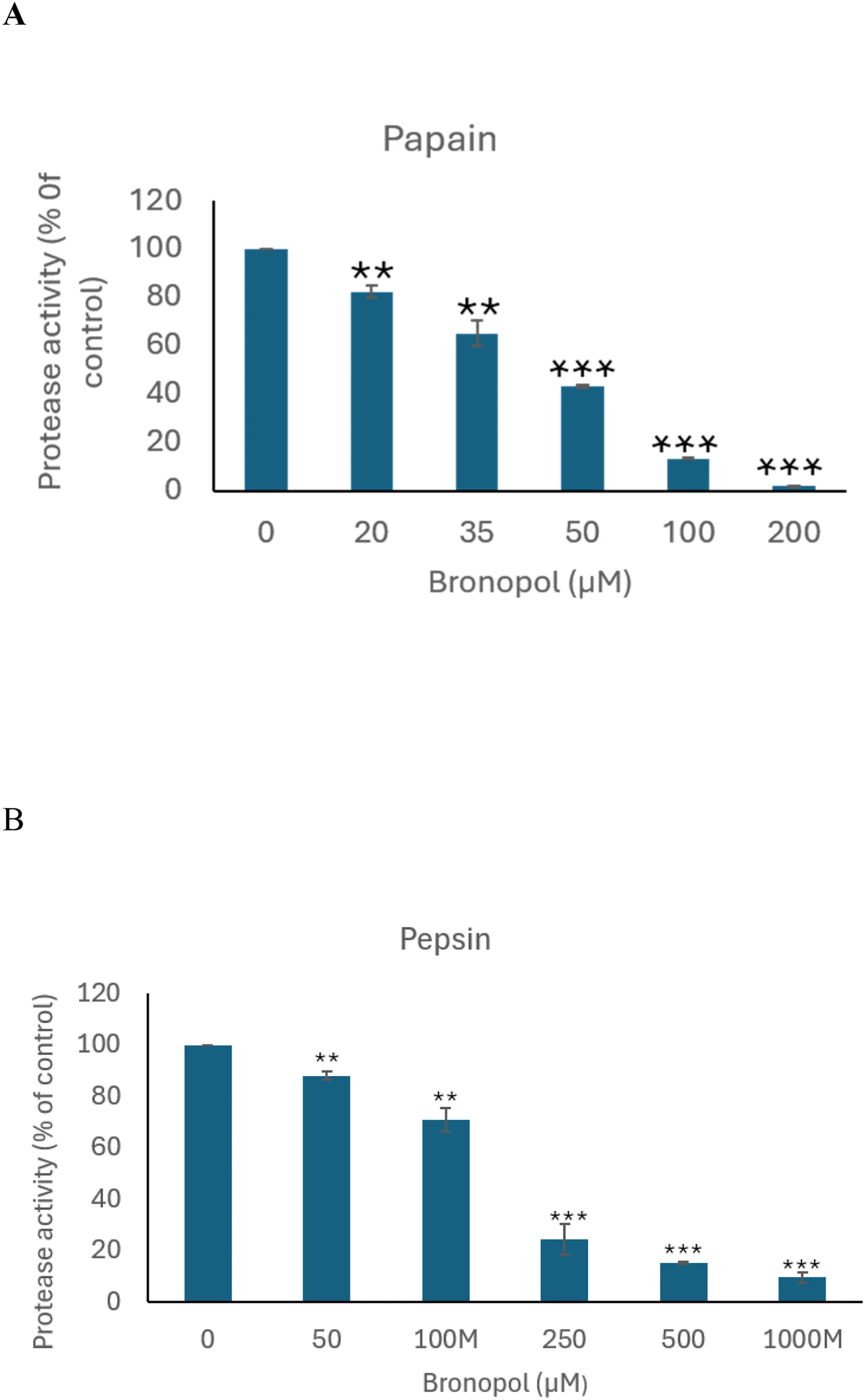
**Dose dependent inhibition of papain and pepsin by BP. A**, Effect of different concentrations of BP on papain activity. **B**, Effect of different concentrations of BP on pepsin activity. Protease activity was measured using the Pierce Protease Assay Kit as described in methods section. Papain and pepsin concentrations in the reaction mixture were 100 μg/mL and 25 μg/mL, respectively. N = 6. *p < 0.05, **p < 0.01, ***<0.001.

Papain, a cysteine protease, was used to study the inhibitory effects of BP on cysteine proteases. The inhibitory effect of BP on papain activity was evaluated over a concentration range of 0–1000 μM. As shown in Fig. 3A, papain activity declined sharply with increasing BP concentrations, indicating strong dose-dependent inhibition. In the absence of BP, papain displayed full activity (100%). At 20 μM, enzyme activity decreased to approximately 82%, and further declined to about 65% at 35 μM. A marked reduction occurred at higher concentrations: papain activity dropped to around 43% at 50 μM and to roughly 13% at 100 μM. At the highest concentration tested (200 μM), papain activity was nearly abolished, reaching only ∼2% of the control level. Overall, these results demonstrate that papain is highly sensitive to BP, exhibiting steep inhibition even at relatively low concentrations. The IC50 value for BP in this assay was calculated to be 47.1 μM (Supplementary material Fig. 2 and Table 1).

### 3.4 Inhibition of aspartic proteases by BP

Inhibitory effects of BP on aspartic proteases were studied using pepsin enzyme. The effect of BP on pepsin activity was examined across concentrations ranging from 0 to 1000 μM. As shown in Fig. 3B, BP produced a gradual but concentration-dependent reduction in pepsin activity. Pepsin retained full activity (100%) in the absence of BP. At 50 μM, enzyme activity decreased modestly to approximately 88%, followed by a further decline to around 71% at 100 μM. indicating relatively limited inhibition at lower concentrations. A more pronounced decline occurred at 250 μM, where activity dropped to roughly 24.6%. Higher BP concentrations resulted in substantial inhibition: activity was reduced to about 15% at 500 μM and further to approximately 9% at 1000 μM. These results indicate that although pepsin is less sensitive to BP at lower concentrations, significant inhibition occurs at concentrations of 250 μM and above. The IC50 value for BP in this assay was calculated to be 125.3 μM (Supplementary material Fig. 2 and Table 1).

### 3.5 Inhibition of proteolytic degradation of bovine serum albumin and ovalbumin by BP

**Fig. 4:**
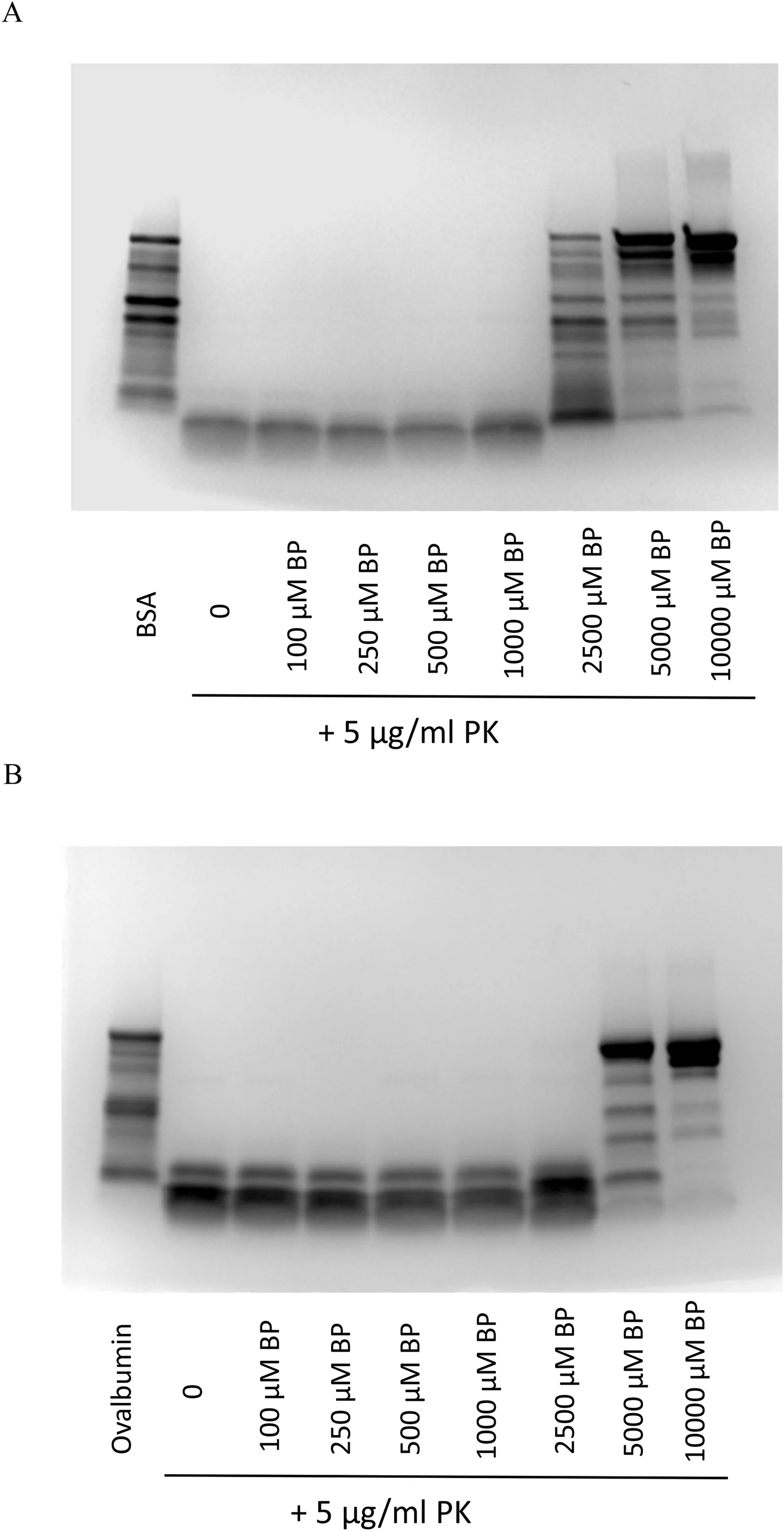
**BP mediated inhibition of proteolytic degradation of BSA and ovalbumin by proteinase k. A**, Effect of BP on proteolytic degradation of BSA by proteinase K. **B**, Effect of BP on proteolytic degradation of ovalbumin by proteinase K. BSA (0.5 mg/mL) and ovalbumin (1 mg/mL) were incubated with proteinase K (5 μg/mL) and varying concentrations of BP at room temperature for 2 days. Reactions were terminated by addition of 5× SDS loading buffer followed by heating at 80 °C for 10 min. Protein cleavage products were analyzed by 4–20% Tricine SDS-PAGE, and gels were stained using GelCode Blue Stain Reagent. Panel A and Panel B are representative results from three independent experiments.

Fig. 4A shows the SDS-PAGE analysis of bovine serum albumin (BSA) following proteolytic digestion by proteinase K and the inhibitory effects of BP across a range of concentrations. The leftmost lane contains untreated BSA, displaying the characteristic intact BSA band. In contrast, the lane corresponding to BSA incubated with proteinase K alone shows a near-complete loss of the intact BSA band, with only a single low–molecular-weight fragment visible, indicating extensive proteolysis.

In samples treated with proteinase K in the presence of 100, 250, 500, or 1000 μM BP, the degradation pattern closely resembles that of proteinase K treatment alone. The intact BSA band remains absent or markedly diminished, and a single low–molecular-weight fragment predominates, suggesting that these BP concentrations do not significantly inhibit proteinase K activity.

However, a clear BP-dependent protective effect emerges at higher concentrations (2500, 5000, and 10 000 μM). These lanes show progressive restoration of the intact BSA band and a corresponding reduction in proteolytic fragments. At intermediate concentrations, partial inhibition is evident, while at the highest concentrations the inhibition is nearly complete, as demonstrated by the strong preservation of full-length BSA and minimal formation of low–molecular-weight products.

Overall, Fig. 4A demonstrates that BP inhibits proteinase K–mediated degradation of BSA in a dose-dependent manner, with substantial protection observed only at higher BP concentrations.

Fig. 4B depicts the SDS-PAGE analysis of ovalbumin following proteolytic digestion by proteinase K and the inhibitory effects of BP across a range of concentrations. The leftmost lane contains untreated ovalbumin, displaying the characteristic intact protein bands. In contrast, the lane representing ovalbumin treated with proteinase K alone shows a marked loss of intact protein, with only two low–molecular-weight fragments remaining, indicative of extensive proteolysis.

Samples treated with proteinase K in the presence of 100, 250, 500, 1000, or 2500 μM BP exhibit degradation patterns similar to the proteinase K–only control. The intact ovalbumin bands are substantially diminished, and two prominent low–molecular-weight fragments persist, demonstrating that these BP concentrations do not significantly inhibit proteolysis.

At higher BP concentrations (5000 and 10 000 μM), a clear protective effect becomes evident. These lanes retain strong intact ovalbumin bands, accompanied by markedly reduced or nearly absent low–molecular-weight fragments, indicating substantial inhibition of proteinase K activity.

### 3.6 In silico analyses of interactions between BP and proteases

To understand the mechanism by which BP inhibits protease activities, silico analysis of interactions between BP and proteases were studied. Molecular docking was used to predict whether Bronopol could interact with catalytic or non-catalytic regions of trypsin, proteinase K, papain, and pepsin. Across all enzymes and docking modes, Bronopol showed weak-to-modest predicted docking scores, with top-ranked AutoDock Vina scores ranging from −3.77 to −4.84 kcal/mol (Supplementary material Fig. 3 and Table 1). These values suggest possible low-affinity or transient noncovalent interactions rather than strong stable binding.

For papain, both active-site-focused and blind docking placed Bronopol proximal to the catalytic cleft. The top-ranked active-site and blind docking scores were −3.772 and −3.773 kcal/mol, respectively (Supplementary material Fig. 3 and Table 1). In both poses, Bronopol localized near His159 and adjacent catalytic-cleft residues, supporting a plausible interaction with the papain active-site region (Supplementary material Fig. 3 and Table 1).

For pepsin, Bronopol also localized near the catalytic dyad. Active-site docking produced a top score of −4.012 kcal/mol, with Bronopol positioned near Asp32 and Asp215. Blind docking produced a slightly stronger score of −4.173 kcal/mol and independently localized Bronopol near the same catalytic dyad region. This concordance between blind and active-site-focused docking supports the pepsin catalytic cleft as a plausible Bronopol interaction site (Supplementary material Fig. 3 and Table 1).

For proteinase K, active-site-focused docking produced one of the strongest catalytic-site-proximal scores, with a top Vina score of −4.538 kcal/mol. Bronopol was positioned near Ser224 and His69, two residues of the catalytic triad. Blind docking also localized Bronopol near the catalytic region, with a score of −4.368 kcal/mol and proximity to Ser224 and His69. These results suggest that Bronopol can be accommodated within or adjacent to the proteinase K catalytic cleft (Supplementary material Fig. 3 and Table 1).

For trypsin, the initial active-site docking based on canonical residue numbering was excluded because the selected trypsin PDB structure used different local residue numbering. After identifying the PDB-local catalytic triad as His40/Asp84/Ser191, corrected active-site docking placed Bronopol near Ser191 and His40, with a top Vina score of −4.372 kcal/mol. In contrast, blind docking produced the strongest numerical score among all runs, −4.836 kcal/mol, but the top-ranked pose was located distal to the catalytic triad. Thus, Bronopol can be accommodated near the trypsin catalytic site under active-site-focused docking, but blind docking did not indicate preferential localization to the trypsin catalytic pocket (Supplementary material Fig. 3 and Table 1).

Overall, these docking results suggest that Bronopol can occupy catalytic-cleft-proximal regions of multiple proteases, particularly papain, pepsin, and proteinase K, where blind and active-site-focused docking were concordant. For trypsin, active-site accommodation was observed only after corrected active-site targeting, while blind docking favored a distal non-catalytic region. Because the predicted scores were modest, these findings should be interpreted as putative interaction-site predictions rather than definitive evidence of high-affinity binding or direct catalytic inhibition.

## 4 Discussion

This study identifies a previously unreported functional property of BP’ s ability to inhibit proteases across multiple mechanistic classes. Although BP is widely used as an antimicrobial preservative, its impact on enzyme systems beyond microbial targets has not been systematically explored. Our findings demonstrate that BP exerts inhibitory activity against representative serine (trypsin, proteinase K), cysteine (papain), and aspartic (pepsin) proteases, with inhibition patterns that differ markedly among enzyme classes.

BP produced clear dose-dependent inhibition of all enzymes tested, but the magnitude of inhibition varied. Papain was the most sensitive, showing near-complete loss of activity at 250–1000 μM. This heightened susceptibility may reflect the nucleophilic cysteine residue in papain's active site, which could react with BP-derived electrophilic species, consistent with BP's known ability to oxidize thiol groups in proteins [3,4,12]. Serine proteases also showed strong inhibition, though higher concentrations were required to achieve comparable loss of activity. Even though proteinase K and trypsin are serine-proteases, both proteins contain redox-sensitive residues (Cys, Met, His) that are structurally important. BP is a strong thiol oxidizer and a moderate one-electron oxidant. Oxidation of structural Cys, Met and His in-proteinase K and trypsin may cause disruption of protein folding and alter the geometry of the active-site catalytic triad (Ser–His–Asp) reducing catalytic efficiency, of serine proteases even though Ser residue itself is untouched. Pepsin, which relies on acidic aspartate residues for catalysis, exhibited the lowest sensitivity at early doses but experienced substantial inhibition above 250 μM. These distinct sensitivity profiles suggest that BP may interact with proteases via multiple mechanisms, potentially involving oxidative modification, crosslinking, or disruption of catalytic residues.

The molecular docking analyses suggest that Bronopol is capable of occupying regions proximal to the catalytic clefts of several structurally diverse proteases, although the predicted interactions were generally weak based on AutoDock Vina scoring. Across all enzymes examined, docking energies ranged from approximately −3.8 to −4.8 kcal/mol, values more consistent with transient or low-affinity noncovalent interactions than with strong stable inhibitor binding. Accordingly, the docking results should be interpreted cautiously as predictive interaction-site models rather than definitive evidence of potent enzymatic inhibition.

Among the proteases evaluated, papain, pepsin, and proteinase K showed the strongest agreement between blind and active-site-focused docking approaches. In papain, Bronopol localized near the catalytic cleft in both docking modes, particularly in proximity to His159, suggesting that the active-site region is sterically accessible to the compound. Similarly, for pepsin, both docking approaches independently positioned Bronopol near the catalytic Asp32/Asp215 dyad, supporting the possibility of interaction within the catalytic groove. Proteinase K demonstrated comparable behavior, with Bronopol localized near Ser224 and His69 of the catalytic triad under both active-site-focused and blind docking conditions. The concordance between targeted and unbiased docking in these enzymes strengthens the interpretation that Bronopol has a reproducible tendency to associate with catalytic-cleft-adjacent regions in multiple protease classes.

In contrast, trypsin displayed less consistent localization behavior. After correction for PDB-specific catalytic residue numbering, active-site-focused docking successfully positioned Bronopol near His40 and Ser191, indicating that the catalytic cleft can accommodate the molecule. However, blind docking identified a distal non-catalytic binding region as the energetically preferred site despite producing the strongest numerical docking score observed in the study. This discrepancy may indicate that Bronopol lacks strong intrinsic specificity for the trypsin catalytic pocket and may instead preferentially associate with alternative surface regions under unconstrained conditions.

Collectively, these findings suggest that Bronopol possesses the structural capacity to transiently associate with catalytic-cleft-proximal regions of several proteases, although the modest predicted affinities imply that any inhibitory effects are unlikely to arise solely from strong reversible binding interactions. Given Bronopol's known chemical reactivity, particularly toward nucleophilic residues such as cysteine or histidine, it is possible that weak initial noncovalent association could facilitate subsequent chemical modification or indirect perturbation of enzyme structure and function. Nevertheless, additional biochemical and kinetic studies would be required to determine whether the predicted docking poses correspond to experimentally relevant inhibitory mechanisms.

The SDS-PAGE analyses using BSA and ovalbumin as natural substrates further confirmed these inhibitory effects. Proteinase K alone rapidly degraded both proteins, whereas BP at 2500–10 000 μM markedly preserved full-length substrates.

The apparent difference between the inhibitory effects of BP observed in the kinetic assay and the SDS-PAGE proteolysis experiments is likely due to substantial differences in experimental design. The kinetic assay used succinylated casein, a modified and highly accessible substrate, under optimal conditions for proteinase K activity (pH 8.5, short incubation), whereas the SDS-PAGE experiments used native proteins (BSA and ovalbumin) under near-physiological conditions (PBS, pH 7.4, room temperature) with prolonged incubation. Although 1000 μM BP reduced proteinase K activity in the kinetic assay, residual proteolytic activity over extended incubation may still have been sufficient to degrade native protein substrates. Additionally, although we did not directly examine this possibility, we speculate that the reduced stability of BP under neutral pH conditions [13] may have contributed to the diminished inhibitory activity observed during prolonged exposure. These factors likely explain why higher BP concentrations were required to achieve measurable substrate protection in the SDS-PAGE experiments.

These findings have several implications. First, they suggest that BP may influence proteolytic stability in formulations where both BP and endogenous or contaminating proteases are present. This could be beneficial in contexts requiring protein integrity—such as diagnostic reagent preservation, enzyme storage, or biomolecular formulation stability. Conversely, unintended inhibition of beneficial proteases in industrial systems or biological assays may require careful consideration when BP is used as a preservative.

The observed inhibitory concentrations, particularly in the millimolar range for complete suppression of proteinase K, indicate that BP is not a high-affinity protease inhibitor. Nonetheless, its cross-class inhibitory and dose-dependent activity may contribute to its antimicrobial effects, as proteases play essential roles in microbial physiology, virulence, and metabolism.

A limitation of this study is that mechanistic insights into BP–protease interactions were not directly examined. Future work employing mass spectrometry, structural analysis, or kinetic modeling will be essential to determine whether BP modifies proteases covalently, disrupts catalytic residues, or induces oxidative damage. Additionally, evaluating BP's effects on a wider panel of metalloproteases, threonine proteases, and glutamic proteases would help determine whether its inhibitory activity extends to all major protease families.

The identification of Bronopol as a protease inhibitor expands its known functional profile beyond its conventional antimicrobial and preservative roles, suggesting potential utility in both industrial and research settings. In biological and biochemical applications, Bronopol may serve as a useful reagent for preserving protein integrity by limiting proteolytic degradation during sample preparation, storage, or processing. Additionally, its protease-inhibitory activity could have relevance in industrial processes where protease control is important, such as biotechnology, pharmaceutical formulation, and protein-based product stabilization. These findings also provide a foundation for further mechanistic studies to better understand the mode of protease inhibition and to evaluate its selectivity and safety in different applications. We have revised the manuscript to more clearly discuss these potential applications and to highlight the broader significance of this work in expanding the functional utility of Bronopol.

In summary, this study reveals that BP functions as an inhibitor of serine, cysteine, and aspartic proteases, with significant inhibitory effects at moderate to high concentrations. These findings expand the functional understanding of BP beyond its established antimicrobial actions and highlight its potential relevance in protein stabilization, industrial enzyme management, and biochemical assay design.

## CRediT authorship contribution statement

**Chao Jiang:** Writing – review & editing, Methodology, Formal analysis, Data curation. **Gary Krzyzanowski:** Writing – review & editing, Validation, Methodology, Formal analysis. **M. Rohan Fernando:** Writing – original draft, Resources, Project administration, Investigation, Funding acquisition, Formal analysis, Conceptualization.

## Generative AI use

This work was edited for English and grammar with assistance from ChatGPT, a generative AI language model developed by OpenAI.

## Funding

This work was supported by 10.13039/100003334The Ryan Foundation under Grant No. PG64311 to MRF. The funding body had no role in the design of the study, data collection, analysis, and interpretation or in writing the manuscript.

## Declaration of competing interest

All authors declare that there is no conflict of interest to report.

## Data Availability

Data will be made available on request.
