# Supplementary material for: Exploring the protease inhibitory potential of 2-bromo-2-nitro-1,3- propanediol (Bronopol): Initial findings
Source: Biochem Biophys Rep. 2026 Sep 17;48:102791. doi: 10.1016/j.bbrep.2026.102791 (PMC13601044; doi:10.1016/j.bbrep.2026.102791)
Supplement: Supplementary file 1 [file mmc1.docx]

**Supplementary Materials**

**Supplementary material Figure 1**

**Supplementary material Figure 1. Effect of BP on reaction between primary amines and TNBSA.** Glycine (200 µg/100 µL) was dissolved in assay buffer and treated with TNBSA in the presence or absence of 1000 µM BP. There was no statistically significant difference in OD 450 nm values for glycine only and glycine with BP. This indicates that BP does not interfere with the reaction between primary amines and TNBSA reaction.

**Supplementary material Figure 2**

**Supplementary material Figure 2. Assay linearity for proteinase K, trypsin, papain and pepsin were done using a dilution series of BP.** Mean percent inhibition ± 95% CI (n = 3). Curve: four-parameter logistic (4PL) fit. Assay linearity for proteinase K, trypsin, papain and pepsin assays show R^2^ value of 0.998, 0.999, 0.999 and 0.996, respectively. Calculated IC50 values for proteinase K, trypsin, papain and pepsin were 126.6, 90.8, 47.1 and 125.3 µM, respectively.

**Supplementary material Table 1**

Table 1. BP dose-response fit parameters (4PL)

| Enzyme | Enzyme conc. | IC_50_ (µM) | Hill slope | Bottom (%) | Top (%) | R^2^ |
| --- | --- | --- | --- | --- | --- | --- |
| Proteinase K | 5 µg/mL | 126.6 | 2.24 | 16.9 | 77.1 | 0.998 |
| Trypsin | 25 µg/mL | 90.8 | 1.96 | 4.1 | 86.7 | 0.999 |
| Papain | 100 µg/mL | 47.1 | 2.33 | 5.4 | 100.9 | 0.999 |
| Pepsin | 30 µg/mL | 125.3 | 2.95 | 6.1 | 85.3 | 0.996 |

Mean % inhibition from triplicate wells (n = 3). Curves fit into a four-parameter logistic (4PL) model, weighted by SEM. IC_50_ is the BP concentration at the midpoint between bottom and top asymptotes. Proteinase K excludes 500 and 1000 µM; Pepsin excludes 1000 µM. Error bars in figures are 95% Cis (Student’s t, df = 2).

**Supplementary material Figure 3**

**Supplementary data Figure 3. Predicted interaction sites between Bronopol and proteases.** Representative top-ranked docking poses are shown for Bronopol against papain, pepsin, proteinase K, and trypsin. Bronopol is shown as cyan sticks, catalytic residues are shown as orange sticks, and nearby residues are shown in pale green. For papain, pepsin, and proteinase K, the displayed poses correspond to docking modes that localized Bronopol near catalytic-site residues. For trypsin, the corrected active-site-focused docking pose is shown using the PDB-local catalytic triad His40, Asp84, and Ser191. Docking scores were weak-to-modest overall and should be interpreted as relative computational predictions rather than experimentally measured binding free energies.

**Supplementary material Table 2**

Table 2. Molecular docking summary for Bronopol against proteases

| Enzyme | Docking mode | Top Vina score (Kcal/mol) | Catalytic site proximal? | Key catalytic or nearby residues | Interpretation |
| --- | --- | --- | --- | --- | --- |
| Papain | Active-site | -3.772 | Yes | His159 | Active-site proximal pose |
| Papain | Blind | -3.773 | Yes | His159 | Blind docking also localized near catalytic cleft |
| Pepsin | Active-site | -4.012 | Yes | Asp32; Asp215 | Active-site proximal pose |
| Pepsin | Blind | -4.173 | Yes | Asp215; Asp32 | Blind docking also localized near catalytic dyad |
| Proteinase k | Active-site | -4.538 | Yes | Ser224; His69 | Active-site proximal pose |
| Proteinase K | Blind | -4.368 | Yes | Ser224; His69 | Blind docking also localized near catalytic cleft |
| Trypsin | Active-site corrected | -4.372 | Yes | Ser191; His40 | Corrected active-site-focused pose |
| Trypsin | Blind | -4.836 | No | None | Blind docking favored distal non-catalytic region |

**Table 2. Molecular docking summary for Bronopol against proteases.** AutoDock Vina docking was performed using both active-site-focused and blind docking strategies. The top-ranked Vina score for each docking run is shown in kcal/mol. Poses were classified as catalytic-site proximal when Bronopol was positioned within 5 Å of one or more catalytic residues in the corresponding protein structure. Key residues indicate catalytic or catalytic-proximal residues near the top-ranked Bronopol pose. For trypsin, the corrected active-site-focused docking result is reported using the PDB-local catalytic triad His40/Asp84/Ser191; the initial active-site run based on canonical numbering was excluded because the selected trypsin structure used different residue numbering. Vina scores should be interpreted as relative docking scores rather than experimentally measured binding free energies.
